# Efficacy of Dialectical Behavior Therapy (DBT) and DBT-Informed Interventions for Binge Eating in Adolescents: A Systematic Review

**DOI:** 10.7759/cureus.108231

**Published:** 2026-05-04

**Authors:** Mohammed A Khormi, Ali E Abu Hayyah, Wassal F Aljohani, Khalid A Mahasi, Sarah H Shok, Raneem A Bajobeir, Rana A Sumayli, Wojood M Alahmadi, Abdulrahman A Alsini, Obaid F Alharthi, Shahad M Alsubaie, Mohamed A Homoq

**Affiliations:** 1 Family Medicine, Jazan Health Cluster, Jazan, SAU; 2 Emergency Department, Sabia General Hospital, Jazan, SAU; 3 College of Medicine, Taif University, Taif, SAU; 4 College of Medicine, Jazan University, Jazan, SAU; 5 College of Medicine, Vision College, Riyadh, SAU; 6 College of Medicine, King Abdulaziz University, Jeddah, SAU; 7 College of Medicine, Bisha University, Bisha, SAU; 8 College of Medicine, University of Jeddah, Jeddah, SAU; 9 College of Medicine, Universiti Sains Malaysia, Penang, MYS

**Keywords:** adolescents, anorexia nervosa binge-purge subtype, binge eating, binge-eating disorder, bulimia nervosa, cognitive behavioral therapy for eating disorders, dialectical behavior therapy, eating disorders, emotion dysregulation, family-based treatment

## Abstract

Standard treatments for adolescent eating disorders, such as family-based treatment (FBT), may be insufficient for patients presenting with severe emotion dysregulation and co-occurring pathologies. Dialectical behavior therapy (DBT) targets affect regulation and may therefore offer a viable alternative or adjunctive intervention. This study aimed to evaluate the efficacy of DBT and DBT-informed interventions, including those integrated with FBT, for adolescents with eating disorders characterized by binge eating episodes. A systematic literature search was conducted across four databases in accordance with Preferred Reporting Items for Systematic Reviews and Meta-Analyses (PRISMA) guidelines. Studies were included if they involved adolescents with eating disorders characterized by binge eating who received DBT-based interventions. The primary outcome was change in objective binge episodes (OBEs), while secondary outcomes included body mass index (BMI), purging frequency, and Eating Disorder Examination (EDE) measures. Data were analyzed using random-effects meta-analysis. Six studies comprising 102 adolescents were included. Pooled analysis demonstrated a statistically significant reduction in OBEs from pre- to post-treatment (mean difference [MD] = -2.15, 95% confidence interval [CI]: -3.93 to -0.38, p = 0.018). Significant improvements were also observed in vomiting episodes (MD = -2.04, 95% CI: -2.72 to -1.35, p < 0.001), as well as EDE Questionnaire (EDE-Q) global scores (p < 0.001) and EDE purging subscale scores (p = 0.008). In contrast, no statistically significant change was observed in BMI (p = 0.064). Subgroup analyses indicated that interventions integrating DBT with FBT (MD = -2.60, p = 0.044) and those delivered in partial hospital settings were associated with significant reductions in binge eating, whereas outpatient settings did not reach statistical significance in pooled estimates. Overall, DBT and DBT-informed interventions are associated with significant reductions in binge eating and purging behaviors in adolescents with eating disorders, without producing significant changes in BMI. The integration of DBT with FBT appears particularly promising for complex clinical presentations; however, larger randomized controlled trials are needed to confirm these findings and evaluate long-term efficacy.

## Introduction and background

Eating disorders (EDs) are serious psychiatric conditions that typically emerge during childhood or adolescence and are associated with significant medical morbidity, psychological impairment, and elevated mortality risk [1,2]. Early onset is common, with adolescence representing a particularly vulnerable developmental period due to rapid biological, psychological, and social changes that may contribute to disordered eating behaviors and impaired emotion regulation. EDs in this age group are frequently accompanied by psychiatric comorbidities, including depression, anxiety disorders, obsessive-compulsive symptoms, and self-injurious behaviors, all of which contribute to increased clinical complexity and poorer prognosis if left untreated [3,4].

Among ED presentations in adolescents, bulimia nervosa (BN) and binge-eating disorder (BED), as well as binge-purge subtypes of anorexia nervosa (AN-BP), are characterized by recurrent binge eating episodes, often accompanied by compensatory behaviors such as self-induced vomiting, laxative misuse, or excessive exercise [1-3]. These behaviors are associated with significant physical complications, including electrolyte imbalance, gastrointestinal disturbances, and metabolic dysregulation, as well as marked psychosocial impairment [1,5]. Epidemiological studies suggest that BN affects approximately 1-2% of adolescents, while subthreshold binge eating behaviors are considerably more prevalent and may represent early stages of full-threshold disorders or clinically significant conditions in their own right [1-3].

Family-based treatment (FBT) is currently recommended as the first-line intervention for adolescent EDs, particularly for anorexia nervosa and BN, and is endorsed by clinical guidelines such as those from the National Institute for Health and Care Excellence (NICE) [3,5]. FBT emphasizes parental involvement in restoring healthy eating patterns and weight restoration while gradually transferring control of eating back to the adolescent. Although FBT demonstrates strong evidence for many adolescents, treatment response is not universal, and a clinically important subgroup fails to achieve full remission or continues to experience persistent binge-purge behaviors [3,5].

Cognitive behavioral therapy for eating disorders (CBT-ED) is another evidence-based approach and is often used when FBT is not effective or feasible [4,5]. However, CBT-ED may have limited efficacy in adolescents with high levels of affective instability, impulsivity, or comorbid self-harming behaviors, which are known to interfere with treatment engagement and outcomes [4-6]. These limitations highlight the need for alternative or adjunctive interventions targeting underlying psychological mechanisms.

Emotion dysregulation has been increasingly recognized as a central transdiagnostic factor in the development and maintenance of binge eating and compensatory behaviors [3,5]. From this perspective, binge eating may function as a maladaptive coping strategy used to regulate intense negative affect or psychological distress [3,5]. This mechanism is particularly relevant in adolescents with co-occurring non-suicidal self-injury or suicidality, where emotional instability contributes to multiple maladaptive behavioral expressions [3,6].

Dialectical behavior therapy (DBT), originally developed for borderline personality disorder, is a structured, skills-based psychotherapy designed to improve emotion regulation, distress tolerance, interpersonal effectiveness, and mindfulness [3,5]. Given its theoretical focus on reducing maladaptive behaviors driven by emotional dysregulation, DBT has been adapted for use in EDs, particularly those involving binge eating and purging behaviors. DBT-informed interventions, including adaptations integrated with FBT or delivered in outpatient and partial hospitalization settings, aim to address both behavioral symptoms and underlying emotional processes [1,5,6].

Preliminary studies suggest that DBT-based approaches may reduce binge eating episodes and compensatory behaviors in adolescents; however, the evidence base remains limited by small sample sizes, heterogeneous study designs, and a lack of large randomized controlled trials [1-6]. Furthermore, variability in intervention formats, treatment settings, and outcome measures has made it difficult to draw definitive conclusions regarding efficacy.

Given these gaps, there is a need to systematically evaluate the existing evidence on DBT and DBT-informed interventions in adolescent populations with binge eating behaviors. Therefore, the present systematic review and meta-analysis aims to assess the efficacy of DBT-based interventions in reducing objective binge episodes and related ED psychopathology in adolescents, as well as to explore the impact of intervention type, treatment setting, and duration on clinical outcomes.

## Review

Methodology

This systematic review and meta-analysis were conducted in accordance with Preferred Reporting Items for Systematic Reviews and Meta-Analyses (PRISMA) guidelines [7].

Eligibility Criteria

Studies were eligible if they included adolescents aged 12-21 years with EDs involving binge eating, including BN, BED, AN-BP, or subthreshold/mixed presentations. Eligible interventions included DBT or DBT-informed approaches, including DBT combined with FBT, delivered in outpatient, partial hospitalization, or inpatient settings. Studies were required to report pre- and post-treatment data or include a comparator group (e.g., waitlist or active control).

Eligible outcomes were quantitatively reported and included objective binge episodes (OBEs), body mass index (BMI), purging or vomiting frequency, and global ED psychopathology measured using the Eating Disorder Examination Questionnaire (EDE-Q) [8].

Eligible study designs included randomized controlled trials (RCTs), single-arm trials, and observational studies. Studies were excluded if they included only adults, lacked extractable outcome data, or were published as abstracts, case reports, case series, reviews, editorials, letters, or conference proceedings.

Search Strategy

A systematic search was conducted in PubMed, Scopus, Web of Science, and the Cochrane Library. Search terms combined keywords related to DBT, binge eating, ED diagnoses (BN, BED, AN-BP), emotional overeating, and adolescents using Boolean operators. The full search strategy is provided in the supplementary materials.

Study Selection

All records were imported into EndNote for duplicate removal. Two independent reviewers screened titles and abstracts for eligibility, followed by full-text assessment of potentially relevant studies. Disagreements were resolved through discussion or consultation with a third reviewer. The study selection process is presented in a PRISMA flow diagram.

Data Extraction

Data were extracted using a standardized form, including study characteristics (design, country, time frame), eligibility criteria, diagnostic group, outcome assessment tools, intervention details (type, format, setting, duration, and sample size), comparator information, and follow-up duration. Participant-level data included age, sex distribution, BMI, baseline OBEs, and baseline EDE-Q scores.

Risk of Bias Assessment

Risk of bias was assessed according to the study design. Single-arm and observational studies were evaluated using the National Institutes of Health (NIH) Quality Assessment Tool for before-and-after studies without a control group, covering domains such as study population definition, intervention description, outcome measurement validity, follow-up completeness, and statistical analysis quality [9].

For the RCT, risk of bias was assessed using the Cochrane Risk of Bias 2 (RoB 2) tool, which evaluates bias arising from randomization, deviations from intended interventions, missing data, outcome measurement, and selective reporting [10].

Outcome Measures

The primary outcome was the change in OBEs from baseline to post-treatment. Secondary outcomes included changes in BMI, purging or vomiting frequency, and global ED psychopathology measured using the EDE-Q global and purging subscale scores.

Statistical Analysis

All analyses were conducted using R software (R Foundation for Statistical Computing, Vienna, Austria). Effect sizes were calculated as mean differences (MD) with 95% confidence intervals (CI). A random-effects model with inverse variance weighting was used based on extracted means and standard deviations.

Heterogeneity was assessed using the I² statistic and chi-square (χ²) test, with p < 0.05 considered significant. Sensitivity analyses were performed using a leave-one-out approach. Subgroup analyses examined intervention type (DBT vs. DBT + FBT), treatment duration (six months vs. 2.5 months), setting (outpatient vs. partial hospitalization), outcome measurement method (interview vs. questionnaire), and timing of assessment (end of treatment vs. longest follow-up).

Results

Literature Search

**Figure 1:**
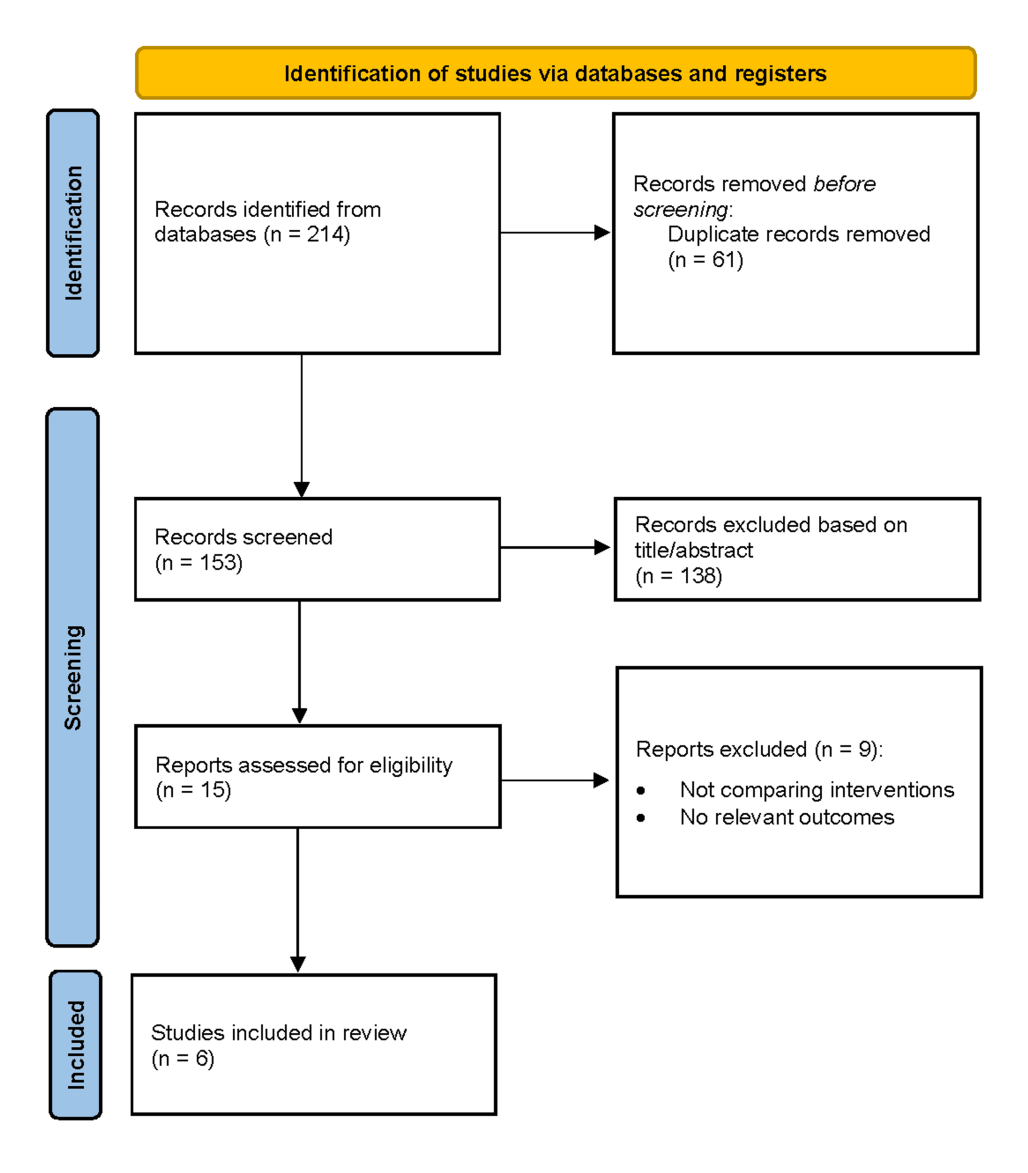
Preferred Reporting Items for Systematic Reviews and Meta-Analyses (PRISMA) flow diagram depicting the study selection process for the systematic review. PRISMA flow diagram detailing the study selection process for this systematic review [7]. Following the identification of records through database searching and other sources, duplicates were removed, and the remaining records were screened. Full-text articles were assessed for eligibility, with exclusions documented along with reasons. Studies meeting the inclusion criteria were included in the final synthesis.

The database search identified 214 records. After removing 61 duplicates, 153 records remained for screening. Following title and abstract screening, 138 records were excluded. Fifteen full-text articles were assessed for eligibility, of which nine were excluded. Ultimately, six studies met the inclusion criteria and were included in the systematic review and meta-analysis (Figure 1).

Characteristics of Included Studies

The six included studies comprised approximately 102 adolescents [1-6]. Four studies were single-arm trials, one was an observational study, and one was an RCT. Studies were conducted in the United States of America, Canada, and Germany. Sample sizes ranged from seven to 40 participants. Participants were aged 15.4 to 16.9 years, and most were female (73%-100%).

Diagnoses included BN, BED, AN-BP, and subthreshold ED presentations. OBEs were assessed using validated instruments, including the Eating Disorder Examination interview (EDE interview), the EDE-Q, and the Structured Interview for Anorexic and Bulimic Disorders (SIAB).

Interventions included standard DBT delivered in individual, group, or combined formats, as well as DBT integrated with FBT. Treatment settings included outpatient, inpatient, day hospital, and partial hospitalization programs. Intervention duration ranged from 2.5 to six months, with follow-up periods up to 24 months (Tables 1, 2).

**Table 1:** Characteristics of studies evaluating DBT and DBT-informed interventions for adolescent eating disorders involving binge eating. This table summarizes the key characteristics of studies included in the systematic review and meta-analysis examining DBT and DBT-informed interventions for adolescents with eating disorders characterized by binge eating behaviors. It includes study design, country, time frame, participant inclusion criteria, diagnostic presentation, instruments used to assess objective binge episodes, intervention type and format, treatment setting, dosage and duration, sample size, comparator conditions (where applicable), follow-up duration, and main study conclusions. Abbreviations: AN = anorexia nervosa; BN = bulimia nervosa; BED = binge-eating disorder; EDNOS = eating disorder not otherwise specified; NSSI = non-suicidal self-injury; DBT = dialectical behavior therapy; FBT = family-based treatment; CBT = cognitive behavioral therapy; SIAB = structured interview for anorexic and bulimic disorders; EDE = eating disorder examination; EDE-Q = eating disorder examination questionnaire; DSM-IV = diagnostic and statistical manual of mental disorders, fourth edition; IQ = intelligence quotient; BMI = body mass index; USA = United States of America.

Study ID	Study design	Country	Time frame	Inclusion criteria	Type of binge eating disorder	Instrument used for assessing objective binge episodes	Intervention type	Format (individual/group)	Setting	Dose	Duration (months)	Number of patients	Comparator	Follow-up (months)	Conclusion
Murray [1]	Single-arm trial	USA	Nov 2012–Jan 2015	Adolescent females with primary diagnosis of BN	BN	EDE-Q	DBT + FBT	Individual and family sessions	Partial hospital program	3–10 hours/day, up to 6 days/week; mean treatment duration 77.18 days	2.5	40	None	2.5	Integration of FBT and DBT shows promise, though mechanisms of symptom remission require further study.
Pennell [2]	Observational study	Canada	2013–2015	Adolescents with eating disorders	AN-BP and BN (subgroup)	Not specified	DBT + FBT	Group and family sessions	Day hospital program	Minimum 6-week program (Mon–Fri); average stay 8.8 weeks	2.03	7 (subgroup)	None	24	DBT-informed day hospital program integrated with FBT is associated with improved outcomes, though implementation challenges remain.
Fischer [3]	Single-arm trial	USA	–	Adolescents aged 14–17; objective binge eating in past 4 weeks; ≥1 suicide attempt or NSSI in past year; height/weight within or above normal range; adolescent assent and guardian participation	BN symptoms or EDNOS with co-occurring suicide attempts and NSSI	Eating Disorder Examination (EDE) interview	DBT	Combined	Outpatient	24 individual + 24 group sessions (weekly over 6 months)	6	10	None	12	Results suggest BN symptoms and other psychopathology can be treated concurrently in adolescents within a single protocol.
Kamody [4]	Single-arm trial	USA	–	Adolescents aged 14–18 endorsing current or prior emotional overeating behaviors	Subthreshold BED behaviors and emotional overeating	Eating Disorder Examination Questionnaire (EDE-Q)	DBT	Group	Outpatient	10 sessions total (including orientation and review); sessions 2–9 were 1 hour each	2.5	30	None	5.5	DBT skills targeting emotionally driven overeating may be useful for subthreshold BED and may prevent progression to full BED.
Salbach [5]	Single-arm trial	Germany	–	Female inpatients with DSM-IV AN or BN confirmed by SIAB; IQ ≥ 85	AN-BP and BN (subgroup)	Structured Interview for Anorexic and Bulimic Disorders (SIAB)	DBT	Combined	Inpatient	2-week program (individual therapy twice weekly + weekly skills group; additional modules included mindfulness and peer sessions)	2.5	14 (subgroup)	None	2.5	Findings are promising and suggest potential benefit of DBT-based inpatient interventions.
Salbach-Andrae [6]	Randomized controlled trial	Germany	–	Females aged 12–21; DSM-IV AN or BN diagnosis; IQ ≥ 85	AN-BP and BN (subgroup)	SIAB	DBT	Combined	Outpatient	25 weeks: 25 individual (~50 min) + 25 skills sessions (~100 min)	5.75	7 (subgroup)	CBT; waitlist control (supportive appointments every 2 weeks, 30 min)	5.75	DBT and CBT both improved outcomes vs waitlist, including reduced distress and improved eating patterns; BMI increased modestly.
Study ID	Study design	Country	Time frame	Inclusion criteria	Type of binge eating disorder	Instrument used for assessing objective binge episodes	Intervention type	Format (individual/group)	Setting	Dose	Duration (months)	Number of patients	Comparator	Follow-up (months)	Conclusion
Murray [1]	Single-arm trial	USA	Nov 2012–Jan 2015	Adolescent females with primary diagnosis of BN	BN	EDE-Q	DBT + FBT	Individual and family sessions	Partial hospital program	3–10 hours/day, up to 6 days/week; mean treatment duration 77.18 days	2.5	40	None	2.5	Integration of FBT and DBT shows promise, though mechanisms of symptom remission require further study.
Pennell [2]	Observational study	Canada	2013–2015	Adolescents with eating disorders	AN-BP and BN (subgroup)	Not specified	DBT + FBT	Group and family sessions	Day hospital program	Minimum 6-week program (Mon–Fri); average stay 8.8 weeks	2.03	7 (subgroup)	None	24	DBT-informed day hospital program integrated with FBT is associated with improved outcomes, though implementation challenges remain.
Fischer [3]	Single-arm trial	USA	–	Adolescents aged 14–17; objective binge eating in past 4 weeks; ≥1 suicide attempt or NSSI in past year; height/weight within or above normal range; adolescent assent and guardian participation	BN symptoms or EDNOS with co-occurring suicide attempts and NSSI	Eating Disorder Examination (EDE) interview	DBT	Combined	Outpatient	24 individual + 24 group sessions (weekly over 6 months)	6	10	None	12	Results suggest BN symptoms and other psychopathology can be treated concurrently in adolescents within a single protocol.
Kamody [4]	Single-arm trial	USA	–	Adolescents aged 14–18 endorsing current or prior emotional overeating behaviors	Subthreshold BED behaviors and emotional overeating	Eating Disorder Examination Questionnaire (EDE-Q)	DBT	Group	Outpatient	10 sessions total (including orientation and review); sessions 2–9 were 1 hour each	2.5	30	None	5.5	DBT skills targeting emotionally driven overeating may be useful for subthreshold BED and may prevent progression to full BED.
Salbach [5]	Single-arm trial	Germany	–	Female inpatients with DSM-IV AN or BN confirmed by SIAB; IQ ≥ 85	AN-BP and BN (subgroup)	Structured Interview for Anorexic and Bulimic Disorders (SIAB)	DBT	Combined	Inpatient	2-week program (individual therapy twice weekly + weekly skills group; additional modules included mindfulness and peer sessions)	2.5	14 (subgroup)	None	2.5	Findings are promising and suggest potential benefit of DBT-based inpatient interventions.
Salbach-Andrae [6]	Randomized controlled trial	Germany	–	Females aged 12–21; DSM-IV AN or BN diagnosis; IQ ≥ 85	AN-BP and BN (subgroup)	SIAB	DBT	Combined	Outpatient	25 weeks: 25 individual (~50 min) + 25 skills sessions (~100 min)	5.75	7 (subgroup)	CBT; waitlist control (supportive appointments every 2 weeks, 30 min)	5.75	DBT and CBT both improved outcomes vs waitlist, including reduced distress and improved eating patterns; BMI increased modestly.

**Table 2:** Baseline characteristics of the included studies. This table presents the baseline demographic and clinical characteristics of participants included in the studies evaluating DBT and DBT-informed interventions for adolescents with eating disorders involving binge eating behaviors. Data include sample size, age, sex distribution, BMI, preoperative objective binge episode frequency, and baseline EDE-Q global scores where available. Some studies did not report all baseline variables, which are indicated by dashes. Abbreviations: DBT = dialectical behavior therapy; FBT = family-based treatment; CBT = cognitive behavioral therapy; WC = waitlist control; N = number; SD = standard deviation; BMI = body mass index; EDE-Q = eating disorder examination questionnaire.

Study ID	Group	Number (N)	Age (years), mean (SD)	Female, N (%)	BMI (kg/m²), mean (SD)	Preoperative objective binge episodes, mean (SD)	Preoperative EDE-Q global score, mean (SD)
Murray [1]	DBT + FBT	35	15.7 (1.11)	35 (100)	26.3 (2.34)	4.03 (6.69)	4.18 (1.8)
Pennell [2]	DBT + FBT	7	15.43 (1.47)	7 (100)	–	–	–
Fischer [3]	DBT	7	16.2 (1.03)	7 (100)	31.2 (6.96)	9.29 (8.9)	2.83 (1.74)
Kamody [4]	DBT	15	15.4 (1.3)	11 (73.3)	–	3.67 (4.95)	–
Salbach [5]	DBT	14	16 (1.6)	14 (100)	19.45 (4.86)	–	–
Salbach-Andrae [6]	DBT	7	16.9 (1.7)	7 (100)	–	–	–
Salbach-Andrae [6]	CBT	11	–	11 (100)	–	–	–
Salbach-Andrae [6]	WC	6	–	6 (100)	–	–	–

Risk of Bias Assessment

Risk of bias in single-arm and observational studies, assessed using the NIH tool, rated two studies as good quality and three as fair quality. The RCT showed some concerns regarding the randomization process, but was overall judged to have a low risk of bias (Table 3).

**Table 3:** Risk of bias assessment of included studies using the NIH tool for single-arm and observational studies and the RoB 2 tool for randomized controlled trials. This table presents the risk of bias assessment of studies included in the systematic review. Single-arm trials and observational studies were assessed using the NIH quality assessment tool, while the RCT was assessed using the RoB 2 tool [9,10]. For NIH-assessed studies, domain-level judgments reflect key methodological criteria including participant selection, intervention description, outcome measurement validity, follow-up completeness, and appropriateness of statistical analyses. For the RCT, RoB 2 domains include bias arising from the randomization process, deviations from intended interventions, missing outcome data, outcome measurement, selection of reported results, and overall risk of bias judgment. Overall ratings summarize study quality or risk of bias classification as determined by the respective tools. Abbreviations: NIH = National Institutes of Health; RoB 2 = Cochrane Risk of Bias 2 tool; RCT = randomized controlled trial.

Study ID	Study design	Risk of bias domains	Domain-level judgment summary	Overall rating
Murray [1]	Single-arm trial	NIH criteria (selection, intervention, follow-up, analysis)	Good methodological reporting; some limitations in sample size and follow-up design	Good
Pennell [2]	Observational study	NIH criteria (selection, intervention, outcomes, follow-up, analysis)	Good implementation description; limited control and statistical adjustment	Fair
Fischer [3]	Single-arm trial	NIH criteria (selection, intervention, outcomes, analysis)	Generally well-described intervention and outcomes; limitations in sample size and blinding	Fair
Kamody [4]	Single-arm trial	NIH criteria (selection, intervention, outcomes, analysis)	Adequate reporting and consistency; limited statistical inference and sample size	Fair
Salbach [5]	Single-arm trial	NIH criteria (selection, intervention, outcomes, analysis)	Strong intervention description and outcome measurement; small sample size	Good
Salbach-Andrae [6]	Randomized controlled trial	RoB 2 (randomization, deviations, missing data, outcome measurement, reporting)	Some concerns due to randomization process; other domains at low risk	Low (some concerns)

Primary Outcome

Three studies were included in the meta-analysis of OBEs. DBT-based interventions were associated with a statistically significant reduction in OBEs (MD = −2.15, 95% CI: −3.93 to −0.38, p = 0.018; I² = 0%). Leave-one-out sensitivity analysis confirmed the robustness of the findings. The effect remained significant when excluding Fischer et al. (MD = −2.08, 95% CI: −3.88 to −0.28, p = 0.024) and Kamody et al. (MD = −2.71, 95% CI: −5.17 to −0.26, p = 0.030), but became non-significant when excluding Murray et al. (MD = −1.72, 95% CI: −4.22 to 0.78, p = 0.178).

Subgroup analyses showed that DBT alone did not produce a statistically significant effect (MD = −1.72, 95% CI: −4.22 to 0.78, p = 0.178), whereas DBT combined with FBT demonstrated a significant reduction in OBEs (MD = −2.60, 95% CI: −5.13 to −0.07, p = 0.044). By treatment duration, 2.5-month interventions showed a significant reduction in OBEs (MD = −2.08, 95% CI: −3.88 to −0.28, p = 0.024), while six-month interventions did not reach significance (MD = −4.72, 95% CI: −15.31 to 5.87, p = 0.389). By setting, partial hospitalization programs showed a significant effect (MD = −2.60, 95% CI: −5.13 to −0.07, p = 0.044), whereas outpatient interventions were not significant (MD = −1.72, 95% CI: −4.22 to 0.78, p = 0.178). By assessment method, EDE-Q-based outcomes showed a significant reduction (MD = −2.08, 95% CI: −3.88 to −0.28, p = 0.024), while EDE interview-based outcomes were not significant (MD = −4.72, 95% CI: −15.31 to 5.87, p = 0.389). At the end of treatment, results remained significant (MD = −2.15, 95% CI: −3.93 to −0.38, p = 0.018), whereas the longest follow-up did not show a significant pooled effect (MD = −4.85, 95% CI: −11.05 to 1.35, p = 0.125).

Secondary Outcomes

No statistically significant change in BMI was observed following treatment (MD = −1.08, 95% CI: −2.22 to 0.06, p = 0.064). A significant reduction in vomiting episodes measured using the SIAB was observed (MD = −2.04, 95% CI: −2.72 to −1.35, p < 0.001). ED psychopathology showed significant improvement following treatment. The EDE-Q global score decreased significantly (MD = −1.91, 95% CI: −2.57 to −1.25, p < 0.001), as did the EDE purging subscale score (MD = −5.47, 95% CI: −9.52 to −1.42, p = 0.008).

Summary of Findings

DBT-based interventions were associated with a statistically significant reduction in OBEs. Significant improvements were also observed in vomiting frequency and ED psychopathology, while no significant change was found in BMI. Subgroup analyses suggested that DBT combined with FBT and interventions delivered in partial hospitalization settings yielded stronger effects. Detailed results are presented in Table 4.

**Table 4:** Summary of findings for DBT and DBT-informed interventions in adolescents with binge eating This table presents pooled effect estimates from the included studies, including primary, secondary, and subgroup analyses. Effect sizes are reported as mean differences (MD) with corresponding 95% confidence intervals (CI) and p-values. Heterogeneity is reported using the I² statistic where applicable. Outcomes include changes in objective binge episodes (OBEs), body mass index (BMI), vomiting frequency, and eating disorder psychopathology measured using the Eating Disorder Examination Questionnaire (EDE-Q) and Structured Interview for Anorexic and Bulimic Disorders (SIAB). Subgroup analyses explore variations in treatment effect according to intervention type (DBT vs DBT + family-based treatment), treatment duration, treatment setting, assessment method, and timing of outcome measurement. Negative mean differences indicate reductions in symptom severity following intervention. Statistically significant findings (p < 0.05) are denoted with an asterisk (*).

Outcome / Subgroup		No. of Studies	Effect Size (MD)	95% CI	p-value
Primary Outcome	Objective Binge Episodes (OBEs)	3	−2.15	−3.93 to −0.38	0.018*
Secondary Outcomes	BMI	3	−1.08	−2.22 to 0.06	0.064
	Vomiting episodes (SIAB)	2	−2.04	−2.72 to −1.35	<0.001*
	EDE-Q Global Score	2	−1.91	−2.57 to −1.25	<0.001*
	EDE Purging Subscale	2	−5.47	−9.52 to −1.42	0.008*
Subgroup Analyses (OBEs)	DBT alone	2	−1.72	−4.22 to 0.78	0.178
	DBT + FBT	1	−2.60	−5.13 to −0.07	0.044*
	Duration: 2.5 months	2	−2.08	−3.88 to −0.28	0.024*
	Duration: 6 months	1	−4.72	−15.31 to 5.87	0.389
	Partial hospitalization	1	−2.60	−5.13 to −0.07	0.044*
	Outpatient setting	2	−1.72	−4.22 to 0.78	0.178
	EDE-Q assessment	2	−2.08	−3.88 to −0.28	0.024*
	EDE interview	1	−4.72	−15.31 to 5.87	0.389
	End of treatment	3	−2.15	−3.93 to −0.38	0.018*
	Longest follow-up	2	−4.85	−11.05 to 1.35	0.125

Limitations

This review has several important limitations. The number of included studies and total sample size were small, which limits statistical power and generalizability. Most studies were single-arm or observational designs without control groups, preventing causal conclusions about the specific effectiveness of DBT compared with other interventions and limiting the ability to rule out nonspecific treatment effects or natural symptom change over time.

There was methodological heterogeneity across studies, including differences in outcome measures (e.g., EDE interview versus EDE-Q) and variability in how binge eating was defined and assessed, which may have contributed to heterogeneity in pooled estimates. In addition, several subgroup analyses were based on single studies or very small subsamples, making these findings exploratory and requiring cautious interpretation.

Finally, the lack of consistent long-term follow-up data limits conclusions regarding the durability of treatment effects on OBEs, compensatory behaviors, and overall ED psychopathology.

## Conclusions

This systematic review and meta-analysis provide preliminary evidence that DBT and DBT-informed interventions may be associated with reductions in OBEs, vomiting, and overall ED psychopathology in adolescents. The integration of DBT with FBT may be particularly relevant for adolescents with complex clinical presentations who do not respond adequately to first-line interventions. However, the current evidence base is limited by small sample sizes and predominantly non-controlled study designs, which restricts the strength of conclusions that can be drawn. Further large-scale, well-designed RCTs are needed to clarify the effectiveness and long-term impact of DBT-based interventions in this population.
